# Legislative initiatives can speed PPPM innovation in medicine

**DOI:** 10.1186/1878-5085-5-S1-A8

**Published:** 2014-02-11

**Authors:** Trevor G Marshall

**Affiliations:** 1 Faculty of Health Sciences, Murdoch University of Western Australia, Autoimmunity Research Foundation, California, USA

## 

Predictive, Preventive and Personalized Medicine (PPPM) not only offers reduced patient suffering, but also a reduction in the proportion of GNP needed for healthcare. Yet there is little public pressure for PPPM. Presses announcements continue to convince patients that the cures they have been promised are just around the corner, and the industry is happy to see things continue this way. Clearly, legislators need to take the lead if there is ever to be any serious exploration of PPPM in Medicine. For example, a decade ago, I and my colleagues developed a detailed understanding of how the Human Microbiome causes chronic disease, especially those diagnoses thought to be ‘autoimmune’. We also retargeted an approved, widely available pharmaceutical to prevent that autoimmune disease from getting worse, and in many case to actually reverse it. Our collaborating physicians have demonstrated success with hundreds of patients - yet some of them have been savagely attacked by their peers. For example, a complaint was laid with the College of Physicians and Surgeons of British Columbia against one of my Canadian colleagues, a coauthor of our peer-reviewed papers, alleging that he wasn’t adhering to “standard of care” during his successful science-based treatment of a patient with refractory, idiopathic, disease. In this case ‘the system’ worked, and his College determined that the physician had properly used a “rationale for the knowledge base and guidelines, accessible in peer-reviewed medical literature, including two papers, from the prestigious “*Annals of the New York Academy of Sciences.*” However, an essentially identical complaint was laid against a physician in Washington State, who was only able to retain her license by promising never to use this new science again, and to have her prescribing activities supervised by a Preceptor. Worse, in Arizona, a practitioner was arrested and charged with “Criminal Child and Vulnerable Adult Abuse.” She has lost her license, and is waiting to hear if she will go to jail. This on substantially the same accusation and evidentiary base as was dismissed in Canada. How are our Universities to focus on ingenious and imaginative scientific solutions when Police are tasked to enforce ‘the standard of care’? Legislators have a responsibility to determine public priorities, and make sure that Industry – all industries, even the Healthcare Industry – are attentive to them. In 2006, California passed simple (and successful) legislation to ensure that a physician or surgeon will not be subject to discipline for suggesting innovative therapies if he/she makes sure the patient is aware of ‘the standard of care’ and gives informed consent. The preamble notes that “it can take up to 17 years for a new best practice to reach the average physician and surgeon, it is prudent to give attention to new developments.” Europe is not immune from the pressures working against innovation, encouragement of pharmacists to second-guess the judgement of physicians has been particularly damaging to the adoption of new therapeutic modalities. Yet the California experience makes it clear that change in Medicine can be promoted with enlightened legislation. Changing the current healthcare focus to one of PPPM will require leadership, and the EU is in the box seat to provide that leadership.

